# Successful treatment of congenital erythropoietic porphyria using matched unrelated hematopoietic stem cell transplantation in an adult: A case report

**DOI:** 10.1002/ski2.342

**Published:** 2024-01-27

**Authors:** Pierre Peterlin, Julia Bonnelye, Alice Garnier, Amandine Le Bourgeois, Thierry Guillaume, Maxime Jullien, Hervé Dutartre, Marie Le Moigne, Caroline Schmitt, Laurent Gouya, Antoine Poli, Sebastien Barbarot, Patrice Chevallier

**Affiliations:** ^1^ Clinical Hematology Nantes University Hospital Nantes France; ^2^ Equipe 12 CRCI2NA ‐ INSERM UMR1307 CNRS UMR 6075 CRCINA IRS‐UN University of Nantes Nantes France; ^3^ Dermatology Department Reference Center for Cutaneous Porphyrias Nantes University Hospital Nantes France; ^4^ Reference Center for Rare Diseases Porphyrias Louis Mourier Hospital AP‐HP, Colombes and Research Center of Inflammation UMR1149 INSERM Université de Paris Paris France

## Abstract

Congenital erythropoietic porphyria (CEP), or Gunther disease, is a rare genetic disease responsible for severe dermatologic, hepatic and/or haematological damages related to the deficient activity of the uroporphyrinogen III synthase. Allogeneic stem cell transplantation (Allo‐SCT) represents the only curative treatment and few allotransplanted cases have been reported in children but not in adults. Here we report for the first time the successful cure of a 46‐year old man with CEP with a 5‐year follow‐up after Allo‐SCT.

## Graphical abstract

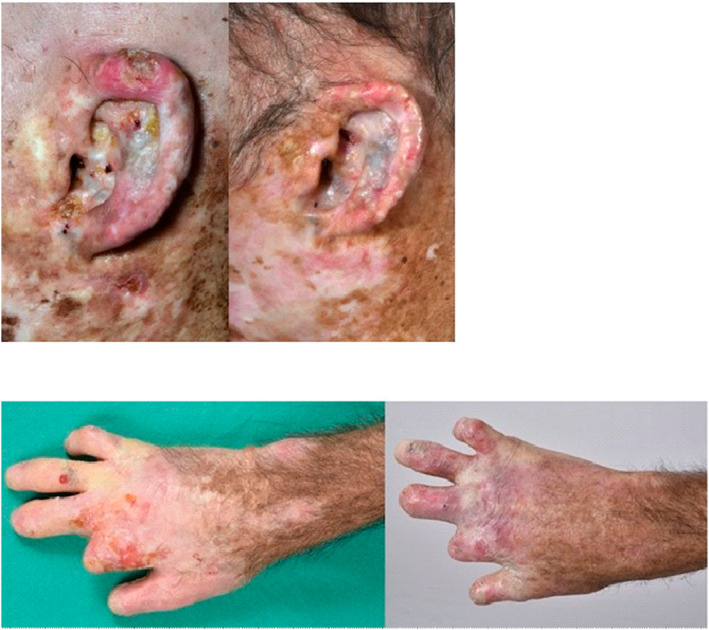

## 1 INTRODUCTION

Porphyrias are a rare group of metabolic disorders caused by the deficient activity of a specific enzyme in the heam biosynthetic pathway.^1^ Among porphyria, the congenital erythropoietic porphyria (CEP), also known as Gunther disease, is the rarest porphyria, with a prevalence estimated at 1 or less per million individuals per year. This congenital autosomal recessive disease is most often due to homozygous or compound heterozygous mutations in the UROIIIS gene on chromosome 10. Genotype/phenotype relationships have been reported, with, for example, increased phenotype severity in carriers of the C73R mutation. Germline mutations in the X‐linked erythroid‐specific transcription factor GATA 1 have been also reported. Mutations in these genes lead to a reduction in erythrocyte UROIIIS enzyme activity (<1–∼10% of the normal), responsible for an accumulation of toxic I‐isomer porphyrins. The depth of the enzyme deficiency modulates the level of porphyrin accumulation and the severity of the disease. Porphyrins first accumulate in erythroid precursors, then are released in plasma by haemolysis, diffuse and accumulate in tissues, mainly in skin and bone marrow but also in liver and dentin.^1^ Porphyrins are eliminated in urine and faeces. Clinical manifestations begin at various ages, ranging from intrauterine (hydrops fetalis) to neonatal, childhood and even adulthood. The most common neonatal manifestations are severe haemolytic anaemia, with liver dysfunction, followed by the appearance of bullous and ulcerating lesions when first exposed to the sun. Severe cases are characterised by extreme photosensitivity, causing skin infections, scarring and mutilations, liver injury, osteoporosis and fractures associated with bone marrow hyperplasia and by haemolytic anaemia which could evolve into pancytopenia, and the need for transfusions and iron chelation, all of this reducing life expectancy.^2^ Symptomatic treatments are ineffective in this serious disease and allogeneic stem cell transplantation (Allo‐SCT) remains the only curative procedure for those eligible. Successful experiences with Allo‐SCT have been reported in children.^3^, ^4^, ^5^, ^6^, ^7^, ^8^, ^9^, ^10^, ^11^, ^12^, ^13^ However, at our knowledge, no such data exist in adults with CEP.

## 2 CASE REPORT

**FIGURE 1:**
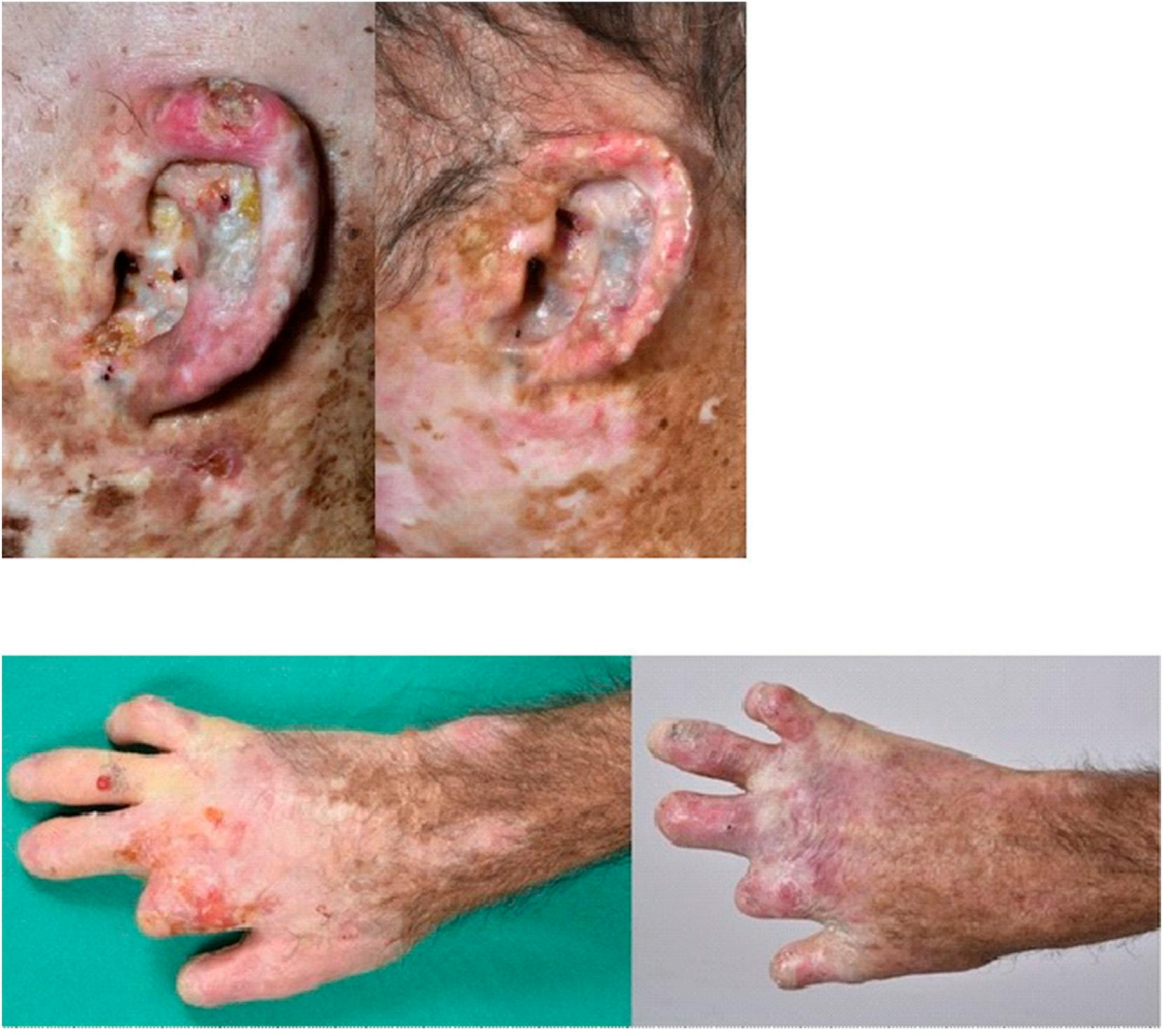
Cutaneous lesions of the ear and the hand before (on the left) and after (on the right) the allograft.

The patient has given its consent to report on his medical case. Institutional approval for the study was obtained as required. The study was conducted in accordance with the Declaration of Helsinki principles. He's a male patient who was diagnosed with CEP at the age of 3 years old. There was no history of CEP in the family. The diagnosis was made in view of the massive accumulation of isomer I porphyrins in red blood cells (in 2016 prior Allo‐SCT 41.1 μmol/L RBC), plasma (2520 nmol/L) and urine (8868 nmol/mmol creatinine, Table 1). Two trans mutations were identified in *UROIIIS* (c.217 T > C p.C73R; c.683C > T p.T228M). Initially, the patient presented with a non‐transfusion‐requiring haemolytic anaemia and, above all, progressively worsening cutaneous and ophthalmological symptoms with severe photosensitivity, diffuse hypopigmented sclerotic plaques, chronic wounds on ears and hands requiring multiple amputations and corneal transplants (Figure 1).

**TABLE 1:** Porphyrin assays in urine, plasma and erythrocyte.

Total porphyrin levels	Before transplant	Day+ 67 post‐transplant	1 year post‐transplant	2 years post‐transplant	5 years post‐ transplant
Urine (nmol/l/mmol creatinine) (normal <30)	8868	308	Not done	Not done	54
Plasma (nmol/L) (normal <20)	2668	100	107	33	21
μmol/LErythrocyte (normal <1.9)	41.1	4.5	1	0.6	0.65

He was addressed in our Haematology Department at age of 44 years old (yo) because of the apparition of a mild pancytopenia associated clinically with asthenia but no tumour syndrome, especially no splenomegaly. Complete blood count showed, as expected, a normocytic regenerative haemolytic anaemia (haemoglobin at 8.6 g/dL), together with mild thrombocytopaenia (100 Giga/L) and neutropenia (1.3 Giga/L). Except the latter, differential was normal. The bone marrow aspiration showed a normal cellularity with no excess of blasts, but ≥20% of erythroid dysplasia without granulocytic or megakaryocytic dysplasia, significant erythroblastosis and signs of hemophagocytosis. Medullary karyotype was normal. A surveillance was decided but few months later the pancytopenia got worse and the patient needed red blood cells transfusions leading to the decision to proceed to Allo‐SCT.

The Allo‐SCT was performed in the Haematology Department in Nantes University Hospital at the age of 46 yo with a matched unrelated donor (no sibling donor available). A myeloablative conditioning regimen (“FB4”) was used consisting of fludarabine 30 mg/m^2^/day (d) (d‐6 to d‐2) + busulfan 3.2 mg/kg/d (d‐6 to d‐3). Graft source was peripheral blood stem cells. Thymoglobuline 2.5 mg/kg/d (d‐2 to d‐1), cyclosporine and mycofenolate mofetil were used as graft‐versus‐host disease (GVHD) prophylaxis. Engraftment was documented at day+30 with a full donor chimaerism and normalisation of complete blood counts. No serious adverse event occurred, especially in terms of infection or liver dysfunction. However, a grade 3 acute GVHD of the gut was documented at day+ 91 which was treated with success with corticosteroids. The patient was free of all systemic immunosuppressive treatments at month+ 7. A mild ocular chronic GVHD developed few months later and persists currently but is controlled by administration of local immunosuppressive eye drops.

We were able to perform various porphyrin assays during the follow‐up after Allo‐SCT (Table 1). Erythroid porphyrin concentration levels decreased drastically in the plasma and the urine as soon as day+ 67 after transplant and have now, after five years, reached quasi normal values. After Allo‐SCT, chromatography of erythrocyte porphyrins shows a major drop in uroporphyrin production, with a normalisation of the proportion of protoporphyrin IX, demonstrating normalisation of the functioning of the haem biosynthesis pathway (Table 2). Indeed, concentrations remains slightly above the normal, suggesting the persistence of an extra‐erythropoiesis porphyrin synthesis which could not be removed by Allo‐SCT. From the clinical point of view, after 5 years, no more skin fragility, excoriation, wound, or chondritis of the nose and ears are present, as shown in Figure 1. Pain has disappeared, sclerotic skin lesions reduced with no discomfort anymore. Photosensitivity disappeared and the patient is now able to swim and to wear t‐shirt. ‘It's a new life!’, as he said. The patient is now considered cured of his disease and has a normal life that he can share with his wife and his two daughters.

**TABLE 2:** Erythrocyte porphyrins chromatography.

Date	Total porphyrin levels in erythrocyte	High performance liquid chromatography						
		Protoporphyrin IX	Harderoporphyrin	Coproporphyrin	Pentacarboxyporphyrin	Hexacarboxyporphyrin	Heptacarboxyporphyrin	Uroporphyrin
	*N* < 1.9 μmol/L erythrocyte	*N*: 64%–92%	*N*: 0%–5%	*N*: 5%–16%	*N*: 0%	*N*: 0%–2%	*N*: 0%–4%	*N*: 0%–7%
Before transplant 01/02/2016	41.1	8.8	0.9	15.5	4.0	5.6	6.1	59.1
Day+ 67 post‐transplant *05/07/2017	4.5	80.2	6.5	4.7	0.0	0.0	0.0	8.6
5 years post‐ transplant*11/02/2022	0.65	71.1	0.0	18.6	0.0	0.0	0.0	10.3

## 3 DISCUSSION AND CONCLUSION

ASCT have been reported for the first time for CEP in 1991 by Kauffman et al^5^ in a 10‐yo female who received a bone‐marrow graft from an HLA‐identical sibling donor. This patient unfortunately died from cytomegalovirus infection after transplant but an encouraging haematologic response was documented. Since then, multiple other paediatric cases have been published showing the efficacy of ASCT to cure CEP.^2^, ^4^, ^6^, ^7^, ^8^, ^9^, ^10^, ^11^, ^12^, ^13^, ^14^, ^15^ Interestingly, a tandem sequential liver transplant and Allo‐SCT from the same haploidentical donor has been reported in a child with CEP, this strategy allowing for discontinuation of immune suppression.^13^ This highlights the potential role of Allo‐SCT in patients needing solid transplant to avoid prolonged immunosuppression, risk of infection, or rejection by inducing tolerance of the transplanted solid organ.

In adults, acquired erythropoietic porphyrias secondary to myeloid malignancy, especially myelodysplastic syndrome, are well identified in adults. These patients were found to have normal erythrocyte URO‐synthase activity, and no constitutive *UROIIIS* or *GATA1* mutations.^14^ The success of Allo‐SCT has been reported in only one adult with such acquired CEP secondary to a myelodysplastic syndrome with chromosome 3 alterations.^15^ Conversely, no Allo‐SCT for CEP in adults has been reported so far.

Porphyrin levels of our patient remain above the normal range at distance of Allo‐SCT, reflecting the porphyrin production by non‐erythroid tissues. Indeed, UROIIIS deficiency is still present in non‐erythroid tissues (for example liver cells) and can lead to a persistent accumulation of porphyrins despite full replacement of erythroid tissues with HSCT as shown by the erythrocyte porphyrins chromatography. Other studies have described a similar situation.^4^, ^12^ As reported here, plasma porphyrin levels remain below a clinically relevant threshold and did not to impair major clinical improvement of skin lesions.

In conclusion, to our knowledge, this is the first reported and successful case of Allo‐SCT in an adult with CEP. This suggest that Allo‐SCT should be considered in children with non‐severe CEP showing progressive aggravation of the disease at adult age. Allo‐SCT can lead to a significant improvement in skin signs, even in patients with major complications due to a long course of the disease. Of course, the decision must be balanced with the risks of complications related to bone marrow transplantation. However, it has to be kept in mind that CEP is generally associated over time with devastating and irreversible lesions.

## CONFLICT OF INTEREST STATEMENT

None to declare.

## AUTHOR CONTRIBUTIONS

**Pierre Peterlin**: Conceptualization (lead); investigation (lead); methodology (lead); resources (lead); writing—original draft (lead). **Julia Bonnelye**: Writing—review and editing (equal). **Alice Garnier**: Writing—review and editing (equal). **Amandine Le Bourgeois**: Writing—review and editing (equal). **Thierry Guillaume**: Writing—review and editing (equal). **Maxime Jullien**: Writing—review and editing (equal). **Herve Dutartre**: Investigation (equal); writing—review and editing (equal). **Marie Le Moigne**: Writing—review and editing (equal). **Caroline Schmitt**: Investigation (equal); writing—review and editing (equal). **Laurent Gouya**: Investigation (equal); methodology (equal); supervision (equal); writing—review and editing (equal). **Antoine Poli**: Writing—review and editing (equal). **Sebastien Barbarot**: Investigation (equal); supervision (equal); writing—review and editing (equal). **Patrice Chevallier**: Conceptualization (equal); investigation (equal); supervision (equal); writing—review and editing (equal).

## FUNDING INFORMATION

This research received no specific grant from any funding agency in the public, commercial, or not‐for‐profit sectors.

## ETHICS STATEMENT

Not applicable.

## Data Availability

The data underlying this article will be shared on reasonable request to the corresponding author.
